# Paraganglioma: An Unexpected Diagnosis in a Patient With Cerebral Venous Sinus Thrombosis and SARS-CoV-2 Infection

**DOI:** 10.7759/cureus.19565

**Published:** 2021-11-14

**Authors:** Olinda Lima Miranda, André Pereira, Margarida Castro, Nuno Carvalho, David Paiva, Ana Costa, Clarisse Neves, Jorge Cotter

**Affiliations:** 1 Internal Medicine, Hospital Senhora da Oliveira, Guimaraes, PRT; 2 Cardiology, Hospital Senhora da Oliveira, Guimaraes, PRT; 3 Internal Medicine, Hospital Senhora Da Oliveira, Guimaraes, PRT

**Keywords:** hypercoagulable state, malignancy, paraganglioma, sars cov-2, cerebral venous sinus thrombosis

## Abstract

Cerebral venous sinus thrombosis (CVST) is the complete or partial occlusion of the main venous sinuses or cortical veins. The most known risk factors are oral contraceptives, pregnancy, thrombophilias, malignancy and infections.

The SARS-CoV-2 infection has been associated with a hypercoagulable state and there are some reported cases of CVST in SARS-CoV-2 patients. Although infection is one of the possible causes of CVST, it is important to rule out malignancy.

We report a case of a 27-year-old male, with a recent SARS-CoV-2 infection, who went to the emergency department for a severe left occipital headache and was diagnosis with CVST. An etiological study revealed a retroperitoneal mass, compatible with a paraganglioma.

## Introduction

Cerebral venous sinus thrombosis (CVST) is the complete or partial occlusion of the main venous sinuses or cortical veins. Changes in blood stasis and composition, as well as changes in the vessel wall, lead to an imbalance that predisposes to progressive venous thrombosis. Headache is the most common symptom in CVST, and sometimes the only one. Altered vision, aphasia, seizures and coma are less frequent symptoms [1]. When CVST is detected early, with the onset of hypocoagulation and the treatment of the underlying cause, the prognosis is favorable [1,2]. The most known risk factors are oral contraceptives, pregnancy, malignancy, infections, inflammatory diseases, trauma, surgery and other prothrombotic conditions like genetic or acquired thrombophilias [1-5].

It has been described an increased number of venous thromboembolism (VTE) and deep venous thrombosis, but less cases of CVST in SARS-CoV-2 infected patients [2].

Ulivi et al. stated that all risk factors must be considered, unless a very clear etiology is suspected (e.g., direct invasion of a sinus by a local ear infection), as patients may have multiple risk factors for CVST [1].

Lázaro et al. showed that the main causes for CVST were coagulation disorders (24.5% of patients), active malignancy (10%) and infectious diseases (7.4%) [5].

Although the relationship between cancer and venous sinus thrombosis is known, there are few studies supporting it [4,5].

## Case presentation

A 27-year-old male, with a personal history of metabolic dysfunction-associated fatty liver disease, overweight and recent SARS-CoV-2 infection (without hospitalization), went to the emergency department for severe left occipital headache, of sudden onset for one day. The pain did not respond to analgesics, progressed to the frontoparietal region, associated with nausea and an episode of vomiting too. He referred no fever nor visual complaints. On physical examination, he was apyretic, normotensive, with a normal heart rate and presented no neurological deficits. There were no relevant changes in the blood analysis or coagulation study. The cerebral computed tomography (CT) scan documented “spontaneous hyperdensity of the rectus and transverse sinuses on the left, as well as of the posterior aspect of the superior longitudinal sinus by venous sinus occlusion; coexistence of tentorial hemorrhage on the left” and the venous CT confirmed the “presence of venous thrombosis involving the ampoule of Galen, the straight sinus, the left hemitorcula and the left lateral and sigmoid sinuses.” He started hypocoagulation and was admitted for surveillance and etiological study.

**Figure 1:**
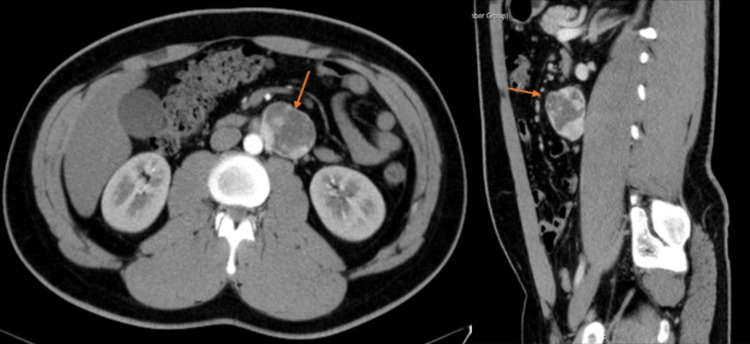
CT scan showing the retroperitoneal mass (arrow)

Regarding the etiological study, complete blood count and peripheral blood smear were normal, HIV and HCV serologies were negative; the prothrombotic study revealed a positive lupus anticoagulant autoantibody result, negative cardiolipin and beta-2-glycoprotein autoantibodies, negative mutation on prothrombin gene and a normal value of factor V Leiden, antithrombin III, Protein S and C. The abdominopelvic CT showed “an uptake mass in the retroperitoneum to the left, laterally to the aorta, in a plane below the emergence of the renal arteries; this mass measures 52 x 35 mm and presents intense and heterogeneous contrast product uptake” (Figure 1); unaltered testicular and thyroid ultrasound.

**Figure 2:**
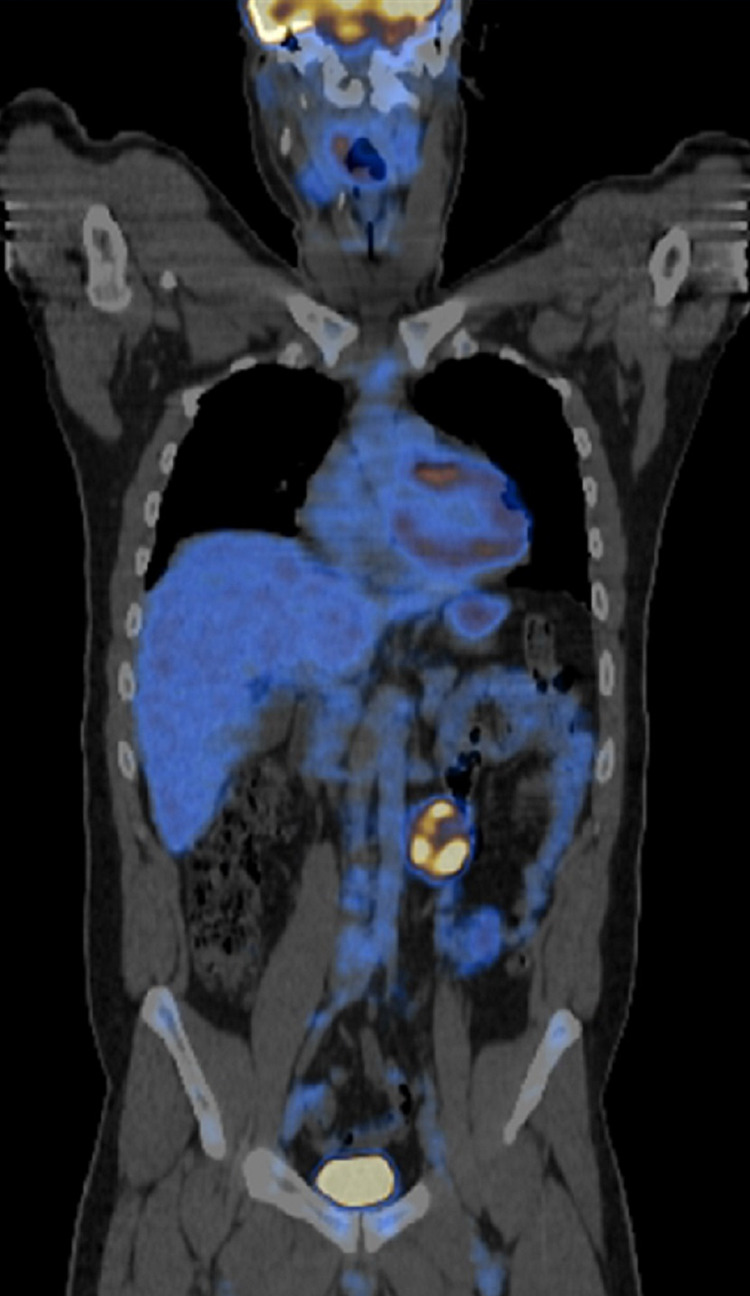
PET-FDG showing a left lateroaortic mass with associated glycolytic hypermetabolism in a coronal plane

**Figure 3:**
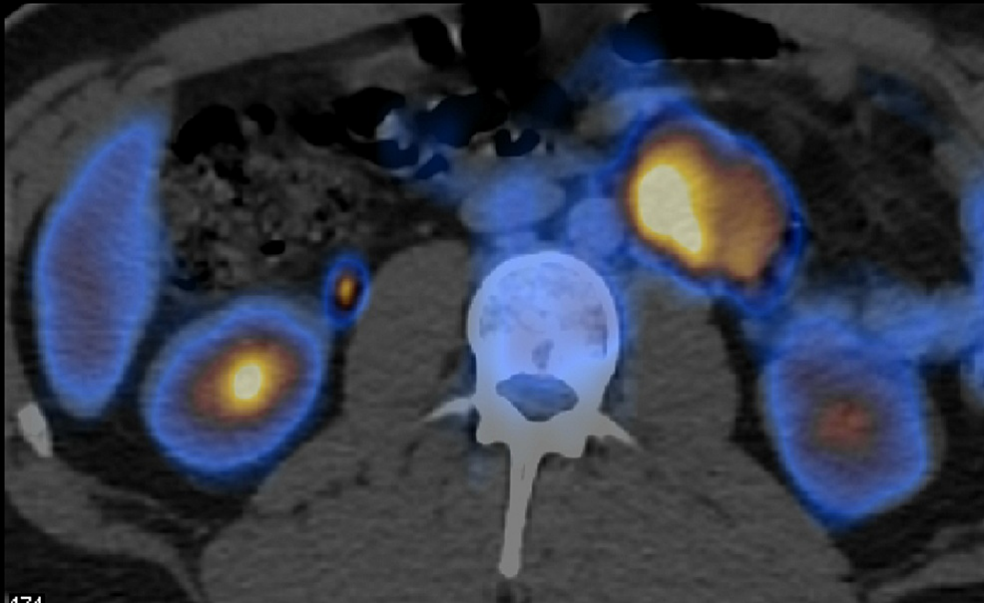
PET-FDG showing a left lateroaortic mass with associated glycolytic hypermetabolism in a transverse plane

Regarding the retroperitoneal mass, a directed study was approached, a fluorodeoxyglucose (FDG) positron emission tomography (PET) was performed, showing “left lateroaortic mass with associated glycolytic hypermetabolism, suggestive of malignant etiology” (Figures 2, 3).

Considering paraganglioma diagnosis, plasma and urinary metanephrines were performed. The results showed positivity for normetanephrine in three urinary samples, for plasma normetanephrine, for noradrenaline and vanilmandelic acid as summarized in Table 1.

**Table 1:** Results for paraganglioma work-up

Catecholamines Work-up	1^st^ sample	2^nd^ sample	3^rd^ sample	Reference range
Urinary Normetanephrine	5,077 µg/24h	2,149 µg/24h	3,786 µg/24h	<444 µg/24h
Plasma Normetanephrine	1,396 pg/mL			<196 pg/mL
Plasma Noradrenaline	1,080 pg/mL			300–650 pg/ml in orthostatic position
Vanilmandelic acid	16.5 mg/24h			<13,6 mg/24h

The patient was referred to a Neuroendocrine Tumor Center and referred to an Internal Medicine consultation to complete a prothrombotic study, posterior to the acute event.

## Discussion

Nowadays, CVST is a well-established complication secondary to SARS-CoV-2 infection [6].

A SARS-CoV-2 infection has been associated with a hypercoagulable state, which is not fully understood; however, some authors propose a mechanism linked to a cytokine storm/cascade of inflammatory events provoked by the viral infection and endothelial damage that leads to the formation of imunothrombus [3,6]. Some markers described in this state were elevation of interleukin-6 (IL-6) (a cytokine with an important role in immune and inflammatory responses) and C-reactive protein (CRP) levels [3].

Although infection may be one possible cause of CVST, it is important to rule out other causes such as genetics (thrombophilia) and malignancy (particularly hematological cancers) [1-4]. In this case, the etiological study found another possible cause of the CVST.

Paraganglioma is a rare neuroendocrine and extra-adrenal tumor, that can cause symptoms due to hypersecretion of catecholamines, such as palpitations, headaches and profuse sweating. The diagnosis is made upon anatomical documentation of the tumor and the increase of plasma-free or urinary fractionated metanephrines. Open resection is recommended for large (>6 cm) or invasive pheochromocytomas to ensure complete tumor resection. Some paragangliomas can become malignant (presence of metastases in nonchromaffin tissue) [7,8].

In the literature, there is also a description of an elevation in IL-6 and other inflammatory markers (CRP and erythrocyte sedimentation rate) in patients with paragangliomas and it is undervalued given the marked effects of catecholamines [9].

Fukumoto et al. demonstrated that patients with pheochromocytoma (another rare neuroendocrine tumor arising from chromaffin cells) may present pyrexia and inflammatory signs as a paraneoplastic syndrome [10].

Silvis et al. showed that a history of cancer increases the risk of CVST, especially in patients with hematological types of cancer compared with those with a solid malignancy [4].

In both pathologies (SARS CoV-2 infection and paraganglioma), increased levels of IL6 were described [3,9]. However, there seems to be no reported case in the literature of the association between CVST and paraganglioma.

## Conclusions

We present a case that shows the importance of excluding all possible causes of CVST if the etiology is not obvious. In this case, we have considered SARS-CoV-2 infection and tumor as possible causes. Diagnosing paraganglioma was very important as it has allowed early surveillance and early treatment in a potentially metastasizing and fatal prognosis.
